# Supplementary material for: Three-Dimensional Microtumor Formation of Infantile Hemangioma-Derived Endothelial Cells for Mechanistic Exploration and Drug Screening
Source: Pharmaceuticals (Basel). 2022 Nov 12;15(11):1393. doi: 10.3390/ph15111393 (PMC9692769; doi:10.3390/ph15111393)
Supplement: Supplementary file 1 [file pharmaceuticals-15-01393-s001.zip › Attachment figure note.pdf]

Figure S1 The cell density of different plate

$*P < 0.05$, compared to the untreated plate, $^{\#}P < 0.05$, compared to the dECM-coated plate

Figure S2 The results of the cell viability after 24 hours of pharmacological intervention

(A) Propranolol, (B) metformin $*P < 0.05$, compared to the control group
