# Supplementary figures and images for: Three-Dimensional Microtumor Formation of Infantile Hemangioma-Derived Endothelial Cells for Mechanistic Exploration and Drug Screening
Source: Pharmaceuticals (Basel). 2022 Nov 12;15(11):1393. doi: 10.3390/ph15111393 (PMC9692769; doi:10.3390/ph15111393)

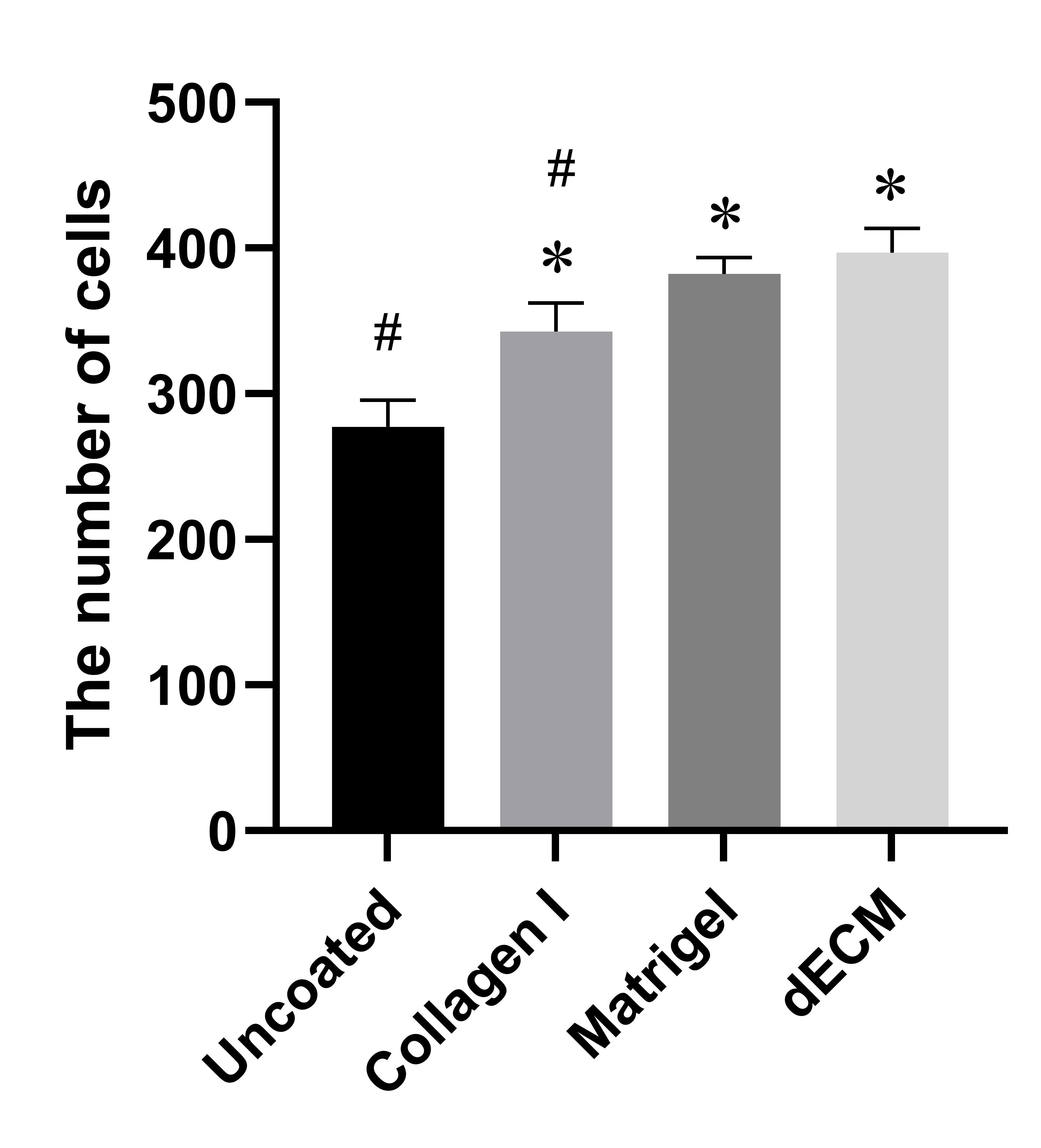

Supplement: Supplementary file 1 [file pharmaceuticals-15-01393-s001.zip › Figure S1.jpg]

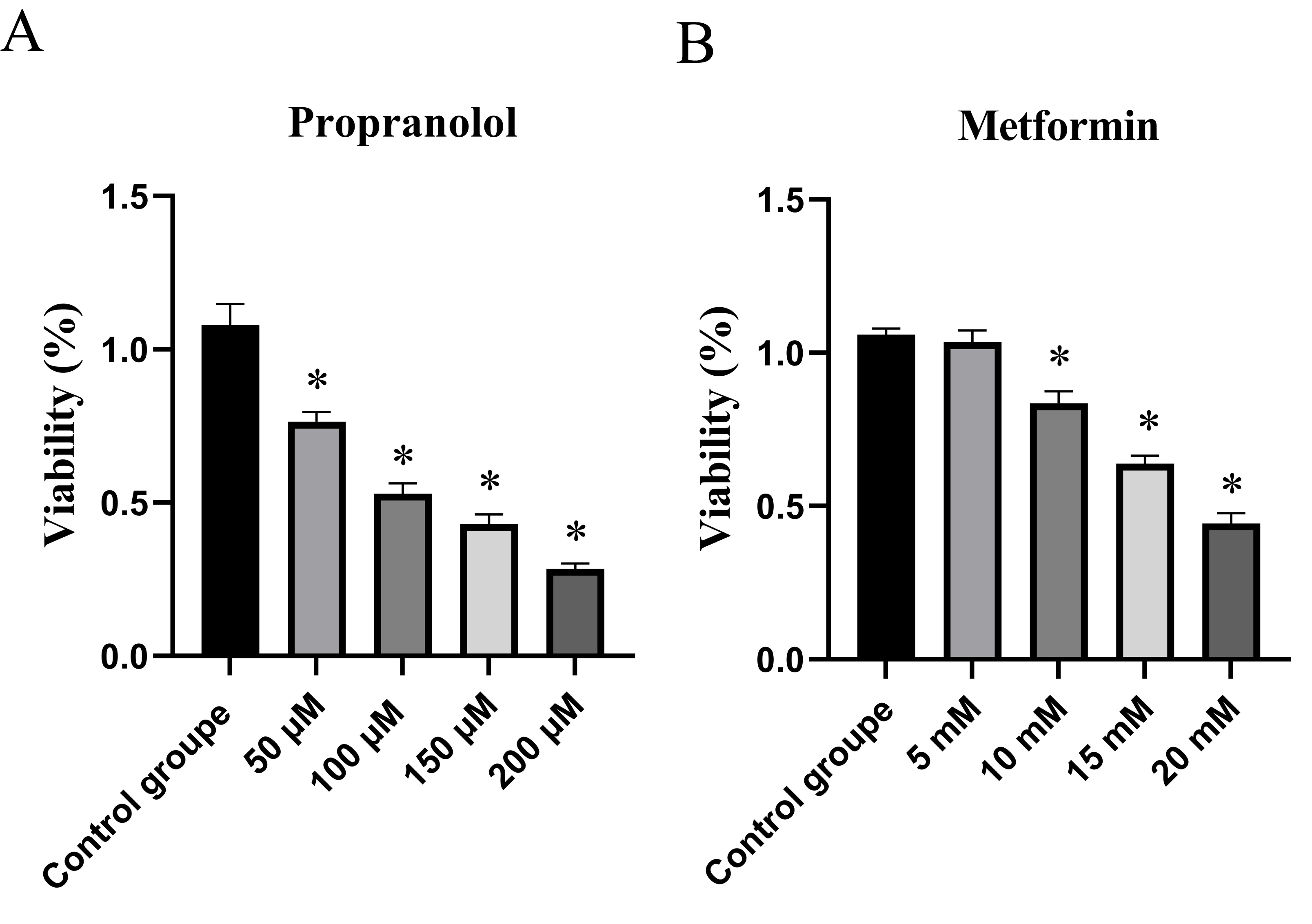

Supplement: Supplementary file 1 [file pharmaceuticals-15-01393-s001.zip › FigureS2.tif]
